# Promoting Healthy Lifestyles in Early Childhood at School with the 0-6 EpPOI Project: Efficacy on Motor Skills and Mediterranean Diet Adherence

**DOI:** 10.3390/nu17132181

**Published:** 2025-06-30

**Authors:** Debora Porri, Elisa La Rosa, Giorgia Pepe, Letteria Anna Morabito, Valentina Arena, Giovanni Luppino, Carla Fazio, Alessandra Li Pomi, Domenico Corica, Angela Alibrandi, Debora Di Mauro, Tommaso Aversa, Malgorzata Wasniewska

**Affiliations:** 1 Department of Human Pathology of Adulthood and Childhood, University of Messina, Via Consolare Valeria, 98124 Messina, Italy; debora.porri@gmail.com (D.P.); arena.valentina95@gmail.com (V.A.); giovilup97@gmail.com (G.L.); fazicarla@gmail.com (C.F.); alessandra.lipomi92@gmail.com (A.L.P.); domenico.corica@unime.it (D.C.); tommaso.aversa@unime.it (T.A.); malgorzata.wasniewska@unime.it (M.W.); 2 Pediatric Unit, “G. Martino” University Hospital, University of Messina, 98124 Messina, Italy; elisalarosa@icloud.com (E.L.R.); letteria.morabito@gmail.com (L.A.M.); 3 Department of Economics, University of Messina, 98100 Messina, Italy; angela.alibrandi@unime.it; 4 Department of Biomedical and Dental Sciences and Morphological and Functional Imaging, University of Messina, 98100 Messina, Italy; debora.dimauro@unime.it

**Keywords:** childhood obesity, fundamental movement skills, Mediterranean diet, preschool, school-based intervention, MOBAK, Kid-Med

## Abstract

Background: Childhood obesity is a global health concern. Early development of fundamental movement skills (FMS) and adherence to the Mediterranean diet (MD) are key modifiable factors for prevention. This study assessed the effectiveness of a multidisciplinary, school-based intervention for childhood obesity prevention. Methods: Children aged 3–5 years from a preschool in Messina, Italy, participated in a 9-month intervention integrating nutritional education and physical activity. FMS were evaluated using the MOBAK test. Anthropometric measurements and MD adherence (through the Kid-Med questionnaire) were collected. Caregivers completed an online survey reporting lifestyle changes. Results: Significant improvements were observed in FMS: object control (score 1) increased from 2.67 ± 1.78 to 4.28 ± 1.82, locomotor skills (score 2) from 4.69 ± 1.96 to 5.83, 5.83 ± 1.70, and total MOBAK score (score 3) from 7.35 ± 3.09 to 10.11± 2.94. (*p* < 0.001 for all). Kid-Med scores significantly improved from (3.79 ± 2.31 vs. 5.03 ± 2.69) (*p* = 0.0027), indicating enhanced MD adherence. Post-intervention, adherence was classified as poor (27.4%), moderate (53.2%), and optimal (19.4%). Although only a minority of parents reported lifestyle changes, over 50% noted increased fruit and vegetable intake in their children. Males showed higher FMS scores and waist circumference compared to females. Conclusions: A school-based multidisciplinary intervention significantly improved motor competence and dietary habits in preschool children. These findings underscore the importance of early, integrated strategies involving families and educators to support healthy development and prevent childhood obesity.

## 1. Introduction

The global burden of childhood obesity continues to rise at an alarming rate, with inadequate nutrition, physical inactivity, and sedentary behaviors recognized as key modifiable contributors to this trend [1,2,3]. In this context, the development of fundamental movement skills (FMS) during early childhood has emerged as a cornerstone of health promotion strategies aimed at reducing obesity risk and fostering lifelong engagement in physical activity [4,5,6]. The preschool years (ages 3–5) represent a critical window for acquiring FMS, which form the foundation for more complex and structured motor patterns used in both recreational and competitive settings [5,7].

Despite the recognition of their importance, FMS are often underdeveloped in young children, particularly in regions where structured physical education programs are limited [8,9]. A growing body of evidence suggests that children with lower physical competence are less likely to be physically active, more prone to excessive weight gain, and less engaged in sports participation over time [10,11,12]. This highlights the urgent need for early interventions that not only enhance FMS but also correct the common misalignment between actual motor proficiency and parental perceptions concerning the importance of healthy lifestyle habits early in age [13,14].

To assess FMS in early childhood, the MOBAK test battery has emerged as a reliable and developmentally appropriate tool for children aged 4 to 8 years, particularly well-suited for those around age 5 [15,16]. The MOBAK test evaluates two core domains of motor competence: object control (e.g., throwing, catching) and self-movement or locomotor skills (e.g., balancing, jumping, sidestepping). Its design enables a standardized and efficient evaluation of children’s movement proficiency in school or clinical settings, making it ideal for large-scale assessments and early screening [15,17]. Low MOBAK scores in 5-year-olds have been consistently associated with decreased physical activity levels, higher body mass index (BMI), and delayed motor development, reinforcing the role of this instrument in identifying children at risk and guiding intervention strategies [16,18].

In our previous study, conducted within the framework of the “0-6 EpPoi-Education to Prevent Childhood Obesity” project, we assessed FMS in preschoolers and identified a significant discrepancy between children’s actual performance and their parents’ estimations [19]. Although more than half of the participating children were enrolled in regular sports activities, only a small fraction reached a satisfactory FMS level, as measured by the MOBAK test battery. Moreover, we observed a notable negative correlation between BMI and locomotor skills, emphasizing the complex interplay between motor competence and body composition in early life [10,19]. In combination with motor development, we also paid attention to nutrition, particularly on Mediterranean Diet (MD) adherence, a pillar of effective childhood obesity prevention [20]. The Mediterranean diet (MD), characterized by high consumption of fruits, vegetables, legumes, whole grains, nuts, olive oil, and moderate intake of fish and dairy, is widely recognized for its health-promoting effects. Originally based on the traditional dietary patterns of countries bordering the Mediterranean Sea, this nutritional model has been associated with reduced risks of cardiovascular disease, obesity, and type 2 diabetes [21]. In children, adherence to the Mediterranean diet has shown significant benefits, including improved body composition, better lipid profiles, and enhanced cognitive development [22,23]. Its emphasis on plant-based foods and healthy fats supports both optimal growth and long-term disease prevention from an early age. Promoting the Mediterranean diet among children not only fosters healthy eating habits but also contributes to sustainable food systems and environmental stewardship [24]. In this context, one of the most used tools to evaluate MD adherence is the Kid-Med questionnaire [25,26]. According to other recent evidence [27,28], we found that adherence to the MD in preschool children was below the optimal level, indicating the urgency of acting also in this field to improve children’s nutrition. High consumption of high-sugar and energy-density snacks and other refined or ultra-processed foods has impacted nutritional habits, and probably these foods have replaced traditional and local Mediterranean nourishment, significantly contributing to an increase in childhood obesity rates [29,30].

Building on these findings, the current study evaluates the impact of targeted school-based interventions designed to promote healthy lifestyles in preschool-aged children. The primary objective of the program was to foster the development of healthy lifestyle behaviors among preschool-aged children and to extend these practices into the home environment through sustained collaboration with parents and caregivers. By comparing data collected before and after implementation of multidisciplinary sessions in a school-based setting, this study aims to assess the effectiveness of these programs in improving FMS outcomes and addressing early indicators of obesity risk, such as MD adherence. Such comparative data are essential for developing evidence-based strategies that engage both children and caregivers in fostering a healthier, more active early childhood environment.

## 2. Materials and Methods

### 2.1. Data Collection

The target population consisted of children aged 3 to 5 years, of both sexes and any ethnic background, along with their families. The present study involved the same cohort of children previously basally examined in our earlier publication [19,20] which was evaluated after 9 months. Leveraging the strong collaboration previously established with a preschool in Messina, a representative and randomly selected sample was recruited from each age group within the specified range. Children were recruited at school during regular school hours, with the collaboration and support of classroom teachers. Recruitment procedures were carried out in coordination with school staff to ensure minimal disruption to educational activities and to facilitate informed parental consent. A comprehensive, multidisciplinary intervention was implemented within the preschool setting, integrating three core components: nutrition education, structured physical activity, and active engagement of both educators and families.

The nutrition education component consisted of age-appropriate, classroom-based instructional modules designed to introduce fundamental concepts of balanced eating. These sessions employed interactive teaching methods—such as visual aids, storytelling, and food-related games—to enhance children’s understanding of dietary variety, the nutritional value of different food groups, and the importance of limiting the intake of processed foods high in sugars, fats, and salt.

In parallel, the intervention incorporated regularly scheduled sessions of structured physical activity, facilitated by trained educators or physical activity specialists. These sessions aimed to promote motor skill development, reduce sedentary behavior, and instill positive attitudes toward movement and exercise from an early age.

To ensure alignment between the school and home environments and to reinforce long-term behavior change, the intervention included periodic meetings and workshops with school staff and families. These sessions provided guidance on how to support healthy habits outside of school, encouraged consistent messaging across settings, and fostered a shared responsibility among stakeholders for the children’s health and well-being.

Data collection after the intervention was conducted on prearranged days during regular school hours by a multidisciplinary research team, ensuring methodological consistency and minimizing disruption to the educational routine.

Parental data were obtained through an anonymous online questionnaire administered via Google Forms. Responses were exported to Microsoft Excel for subsequent analysis. Prior to participation, all respondents were informed about the study objectives. Completion and submission of the questionnaire constituted implied informed consent. No direct benefits were provided to participants for their involvement in the study. The parents themselves filled out the questionnaire, but it is not possible to trace any missing data or connect the parent with their child, as the questionnaire is anonymous. The first evaluation was reported as T0, while the second evaluation was reported as T1.

### 2.2. Fundamental Movement Skills (FMS)

Children’s FMS was reassessed using the MOBAK test battery [15], which evaluates basic motor skills in preschool-aged children across two domains: object movement and self-movement. The MOBAK test is a standardized tool designed to assess basic motor competencies in children [15]. It focuses on two fundamental domains: object movement (e.g., throwing, catching) and self-movement (e.g., balancing, running, jumping). The test aims to evaluate not only the performance of motor skills but also the children’s ability to apply these skills in meaningful contexts. MOBAK is age appropriate, reliable, and suitable for use in school settings to monitor motor development and support physical education interventions [15]. We used MOBAK-SI, which allows for a standardized and cost-effective assessment of the basic motor skills of children aged 4 to 6 years. The procedures and scoring system were applied following the original guidelines to ensure consistency with the initial assessment. Testing was conducted in a standardized setting during school hours and conducted by an expert in the field of physical activity during childhood.

### 2.3. Anthropometric Measurements

Anthropometric parameters were recorded for each child, including body weight and height, using calibrated instruments. Body weight was measured wearing only clothes and without shoes by using a portable electronic scale; height was measured without shoes by means of a portable stadiometer. BMI was calculated as a ratio between weight in kilograms and height squared in meters. Waist circumference (WC) was measured to the nearest centimeter with a flexible steel tape with children standing with crossed arms and placing the hands on opposite shoulders; waist circumference was measured on the horizontal plane between the lowest portion of the rib cage and the uppermost lateral border of the right ilium. The waist-to-height ratio was calculated as a ratio between waist in centimeters and height in centimeters.

### 2.4. Caregivers Questionnaire and Mediterranean Diet Adherence

An anonymous online questionnaire, administered through Google Forms, was used to collect data from parents. In addition to demographic and lifestyle questions, the Kid-Med Questionnaire was re-administered to assess children’s adherence to the MD. Responses were scored according to established guidelines to allow comparison with baseline data.

To assess changes in families’ lifestyle habits, parents were asked to rate, on a 5-point Likert scale (1 = “a little”, 5 = “a lot”), to what extent they had changed their children’s diet or physical activity. This scale allows for the measurement of attitudes or perceptions along a continuum of agreement, providing ordinal-level data that can be used to evaluate trends and levels of consensus among respondents. The complete questionnaire questions are reported in Table 1.

### 2.5. Statistical Analysis

Numerical data (anthropometry and questionnaire results) were expressed as mean, standard deviation (S.D.) and range (minimum and maximum), and categorical variables as absolute frequencies and percentages. These descriptive statistics were calculated for each of the two examined time points of observation. The non-parametric approach was used since the numerical variables were normally distributed (due to the ordered nature of the ordinal variables), as verified by the Kolmogorov-Smirnov test. To perform, for all numerical variables, comparisons between two timepoints, the Wilcoxon test was applied. To evaluate a possible significant correlation between BMI and different scores at t1, the Spearman correlation test was applied. Finally, the comparison of means test was applied to compare the MD adherence expressed as Kid-Med. Statistical analyses were performed using IBM SPSS for Windows, Version 22 (IBM Corp., Armonk, NY, USA). *p*-values lower than 0.05 were considered statistically significant.

## 3. Results

T0 evaluation, which was published elsewhere in two different articles [19,20,27], was part of the “EpPOI” project, aimed at investigating lifestyle habits among preschool children to prevent childhood obesity. Previous findings revealed poor adherence to the Mediterranean diet and high consumption of sweets, despite parental awareness of healthy behaviors. Additionally, previous MOBAK assessments revealed that a significant proportion of children exhibited limited basic motor competencies, highlighting the need for structured physical activity programs in early education settings. Parental perception of children’s physical activity was identified as a significant predictor of better diet quality.

In this evaluation (T1), after a multidisciplinary intervention, 96 Caucasian children were enrolled; 49 were males and 53 were females. Compared to the first assessment (T0) [ref] 21 children were absent from school, and it was not possible for them to repeat the MOBAK test. Table 2 reports anthropometric characteristics of children, expressed as the mean ± standard deviation.

### 3.1. Mediterranean Diet Adherence

Unlike the previous evaluation T0 [19,20], parents’ adherence to the project was lower: in the initial evaluation (T0), 96 subjects completed the survey, whereas in the current study only 62 responded. As in the previous case, all respondents were mothers. Although only 12.9% of mothers declared to have significantly changed their child’s diet, more than half of them (53.2%) declared an increase in their child’s fruit consumption, and 50% of respondents reported an increase also in children’s vegetable consumption. 82.3% of mothers reported that their child has learned useful lessons from previous meetings with specialists, and 61.3% considered nutrition during childhood a very important topic, giving it a score of 5 on a Likert scale.

In order to evaluate MD adherence, we administered the Mediterranean Diet Quality Index for children and adolescents (Kid-Med) questionnaire to the parents, as well as our previous study [20,21,22]. More than half of respondents reached a score which indicates moderate adherence to MD. Table 3 reports results from Kid-Med expressed as frequency and percentage.

The average Kid-Med score expressed as mean ± standard deviation was 5.03 ± 2.69. We performed a comparison of means to assess any statistically significant differences between the previous average score (3.79 ± 2.31) and the one found in this study, and we found a statistically significant difference (*p* = 0.0027).

We also analyzed the responses to each individual question on the Kid-Med questionnaire. Only 21.3% of children skipped breakfast, but 50% of them consumed snacks or sweets for breakfast. Therefore, the percentage of children who consume sweets or candy every day is 48.4%. Answers to the individual Kid-Med items are reported in Table 4.

### 3.2. Functional Movement Skills

Regarding physical activity, a percentage of 17.7% of parents who filled out the questionnaire declared that they had significantly changed their child’s physical activity after the meetings with specialists, and to evaluate FMS, we repeated the MOBAK test on the same children for a total of 96 subjects.

We added the same MOBAK scores from our previous work [19]: *Score 1* for the “object control” section and *Score 2* for the FMS in the “locomotion” section. The sum of the entire questionnaire items was identified as *Score 3*.

Notably, more than half of the children (57.22%) obtained a *Score 3* out of 8 or higher, which corresponds to at least half of the maximum achievable score. However, only 1% of the children achieved the maximum Score 3 out of 16.

Considering also *Score 1* and *Score 2*, 66% of children reached a *Score 1* of 4 or higher, while 70.9% reached a *Score 2* of 4 or higher. The average *Score 1* was 4.28 ± 1.82, while the average *Score 2* was 5.83 ± 1.70, and the sum (*Score 3)* was 10.11 ± 2.94. Interestingly, we found statistical differences in all of the *Scores* between the first and the second evaluation. Results are expressed in Table 5. Considering weight status in the entire sample, we did not find any significant correlation between BMI and the Mobak Scores (*p* < 0.94 for *Score 1*, *p* < 0.12 for *Score 2,* and *p* < 0.377 for *Score 3*).

Finally, we analyzed the sample by dividing it into gender (males and females), and we found statistically significant differences between groups; particularly, males obtained a *Score 1*, *Score 2*, and *Score 3* significantly higher than females (respectively, *p* < 0.007; *p* < 0.016; *p* < 0.002). We also found that males have a WC significantly higher than females (*p* < 0.026).

## 4. Discussion

The present study evaluated the impact of a multidisciplinary, school-based intervention in the context of the EpPOI Project [23] aimed at improving FMS and MD adherence among preschool children in order to act as an early childhood obesity prevention. The findings revealed statistically significant improvements in both motor competencies and dietary patterns, supporting the effectiveness of early interventions in the prevention of childhood obesity.

It is well known that greater FMS competency is associated with higher amounts of physical activity in children and adolescents [31], and this is one established pathway to reducing the risks associated with obesity [32]. An integrated review [33] highlighted that motor function delay appears to be associated with obesity, even if it is not well understood if children with obesity have delayed motor skills as a consequence of their weight or if it is a risk factor for the development of obesity. In our previous evaluation [19], we found a significant negative correlation between BMI and score 2 (locomotor skills), but this correlation was not confirmed in this study, maybe due to a little sample reduction. Improvements in FMS, particularly in object control and locomotor skills as assessed by the MOBAK test battery, confirm existing literature indicating that structured physical activity programs in early childhood significantly enhance motor competence. Notably, our intervention was conducted over a prolonged period of nine months within the school environment, allowing for consistent exposure to motor skill development activities. This long-term approach aligns with previous studies, for example, Veldman et al. (2016) [34], who demonstrated that school-based physical activity interventions lasting at least six months can produce significant improvements in motor skill proficiency among preschool children. Several studies have demonstrated the importance of FMS development in establishing lifelong physical activity habits and reducing obesity risk [35,36]. For instance, Logan et al. (2017) [31] found that preschoolers who participated in targeted movement interventions displayed better motor skills and were more likely to engage in active play over time [31]. Similarly, Bardid et al. (2016) emphasized the importance of age-appropriate interventions in preschool settings to address motor delays and promote equity in health outcomes [35]. Gender differences observed in this study, with males exhibiting significantly higher FMS scores and waist circumference, align with earlier findings. Boys tend to outperform girls in object control tasks, potentially due to biological predispositions, sociocultural expectations, and differential exposure to active play [36]. Legarra-Gorgoñon et al. (2023) reported similar trends in Spanish preschoolers, highlighting the need for gender-sensitive strategies in physical education [37]. In addition, the prevalence of insufficient physical activity in Italy is higher among girls than boys, which could be partly attributed to a lack of FMS development [38].

Regarding nutritional outcomes, the intervention yielded significant improvements in Kid-Med scores, suggesting enhanced adherence to the MD. These results are consistent with school-based nutrition education programs showing positive effects on dietary behaviors among young children [38]. For example, Michael et al. (2024) demonstrated that structured, age-appropriate interventions can improve fruit and vegetable intake and reduce the consumption of sweets in children aged 3–6 years [39].

However, the persistently high consumption of sweets and the suboptimal frequency of fruit and vegetable intake in some children post-intervention indicate that behavior change in this age group is complex and may require longer and more intensive interventions. These findings are echoed in recent literature suggesting that while short-term improvements are achievable, sustaining dietary change often requires ongoing family engagement and reinforcement [40,41,42,43].

Parental involvement emerged as a crucial factor. Although only a minority reported major changes in their children’s physical activity or diet, over half observed increased fruit and vegetable consumption. This reinforces the pivotal role of the home environment in shaping children’s lifestyle behaviors. Piana et al. (2017) similarly observed that parents who actively participated in health promotion initiatives were more likely to adopt healthier routines for their children [44]. Furthermore, intervention studies such as the ToyBox project have highlighted the importance of involving caregivers early to ensure long-term impact [45]. Future interventions should consider incorporating flexible communication methods, personalized feedback, and school-based workshops to increase parental involvement and reinforce health-promoting behaviors at home. Despite increased awareness of healthy eating, many parents face substantial challenges in providing optimal nutrition for their children. One of the most significant barriers is the economic and practical accessibility of food. Ultra-processed foods, which are typically energy dense and nutrient poor, are often more affordable, palatable, and readily available than fresh, minimally processed alternatives [46]. Moreover, these products require little preparation time, making them particularly appealing to time-constrained families.

Economic constraints and food insecurity have been shown to influence dietary patterns, leading families to prioritize quantity and satiety over nutritional quality [47]. This results in greater reliance on low-cost, processed foods high in added sugars, fats, and sodium. Consequently, even when parents are informed about healthy dietary guidelines—such as those underpinning the Mediterranean diet—they may be unable to apply them consistently due to financial and logistical limitations.

Despite the positive outcomes, this study has limitations. First, the absence of a control group limits causal inference. The absence of a control group limits the ability to attribute observed changes directly to the intervention, as external factors (e.g., seasonal variations, maturation, or parental influence) may have influenced outcomes. Without a comparative baseline, it is difficult to determine whether the improvements observed are a result of the intervention itself or due to natural developmental progress or other uncontrolled variables. Second, a key limitation of this study lies in the reliance on self-reported data provided by parents to assess outcomes such as dietary and physical activity changes. Self-report measures are subject to several potential biases, including recall bias, where participants may inaccurately remember or report past behaviors, and social desirability bias, where responses may be skewed toward what is perceived as more acceptable or favorable. These biases can compromise the validity of the reported outcomes, particularly in dietary assessments where precise quantification is essential. While self-reporting remains a practical tool in large-scale studies, especially those involving children, the findings should be interpreted with caution due to these inherent limitations. Third, the intervention’s duration, although sufficient to induce change, may not be long enough to assess sustainability. Future studies should consider a randomized controlled design, include objective dietary assessments (e.g., 24 h recall, biomarkers), and incorporate longer follow-up periods to evaluate long-term effectiveness.

Nevertheless, the current results contribute to a growing body of evidence supporting the implementation of comprehensive, early-life interventions that combine physical activity and nutritional education in preschool settings. The use of validated tools such as the MOBAK and Kid-Med questionnaires strengthens the reliability of the findings and supports their applicability in public health strategies targeting early obesity prevention.

## 5. Conclusions

This study highlights the value of implementing early, multidisciplinary strategies for childhood obesity prevention, targeting multiple determinants of health in an integrated way. The EpPOI intervention, which combined structured physical activity to enhance fundamental movement skills, nutrition education to promote adherence to the Mediterranean Diet, and active involvement of both parents and educators, proved to be effective in improving motor competence, dietary behaviors, and overall health awareness in preschool children. The preschool setting emerged as an ideal environment for educational interventions, offering daily opportunities for reinforcement and continuity between school and home practices. Importantly, the engagement of caregivers and teachers amplified the impact of the intervention, creating a shared culture of health that extended beyond the classroom. Based on our experience, we observed not only statistically significant improvements in motor and dietary outcomes but also qualitative changes in attitudes and behaviors among participating families. These findings suggest that school-based programs should be prioritized within public health policy as scalable, sustainable, and cost-effective tools for early obesity prevention. Investing in this type of early intervention is critical to building long-term resilience against obesity and its comorbidities, and to fostering healthy development trajectories from the earliest years of life.

## Figures and Tables

**Table 1:** Complete questionnaire.

**Question**	**Answer**
1. Are you the mom or the dad?	Mom; Dad
2. Did you modify the eating habits of your child according to the information received?	form 1 [a little] to 5 [a lot]
3. If so, how did you modify your child’s diet?	
4. Did your child increase fruit consumption?	Yes/No
5. Did your child increase vegetables consumption?	Yes/No
6. Do you think nutrition is important in this age group?	form 1 [a little] to 5 [a lot]
7. Did you modify your child’s physical activity according to the information received?	form 1 [a little] to 5 [a lot]
8. If so, how did you modify your child’s physical activity?	
9. Do you think physical activity is important in this age group?	Yes/No
10. Do you think your child has learnt something from the specialists?	Yes/No

**Table 2:** Children’s anthropometric characteristics expressed as mean ± standard deviation.

	**n**	**Mean**	**Standard Deviation**
Age	96	4.69	0.46
Weight	96	20.95	4.30
Height	96	1.12	0.09
Body mass index	96	52.03	6.13
Waist circumference	96	16.62	2.89
Waist-to-height ratio	96	0.46	0.05

**Table 3:** Mediterranean diet adherence (Kid-Med score) expressed as frequency and percentages.

**Adherence**	**Frequency**	**Percentage**
Poor (Kid-Med score ≤ 3)	17	27.4
Moderate (Kid-Med score ≥ 4–7)	33	53.2
Optimal (Kid-Med score ≥ 8)	12	19.4

**Table 4:** Responses to Kid-Med items expressed as frequency and percentage.

**Question**	**Answer**	**Frequency**	**Percentage**
Takes fruits once a day?	No	27	43.5
	Yes	35	56.5
	Missing	0	0.0
Has a second fruit every day?	No	45	72.6
	Yes	17	27.4
	Missing	0	0.0
Has fresh or cooked vegetables regularly once a day?	No	18	29.0
	Yes	43	69.4
	Missing	1	1.6
Has fresh or cooked vegetables more than once a day?	No	43	69.4
	Yes	18	29.0
	Missing	1	1.6
Consumes fish regularly (at least 2–3 times/week)?	No	28	45.2
	Yes	34	54.8
	Missing	0	0.0
Consumes legumes at least once a week?	No	20	32.3
	Yes	41	66.1
	Missing	1	1.6
Consumes fast food more than once a week?	No	44	71.0
	Yes	17	27.4
	Missing	1	1.6
Consumes pasta or rice almost every day? (85 or more times/week)	No	9	14.5
	Yes	53	85.5
	Missing	0	0.0
Takes breakfast regularly?	No	48	77.4
	Yes	13	21.0
	Missing	1	1.6
Has cereal or grains for breakfast?	No	37	59.7
	Yes	25	40.3
	Missing	0	0.0
Has dairy products for breakfast?	No	14	22.6
	Yes	48	77.4
	Missing	0	0.0
Has commercially baked goods or pastries for breakfast?	No	31	50.0
	Yes	31	50.0
	Missing	0	0.0
Consumes nuts regularly? (at least 2–3 times per week)	No	55	88.7
	Yes	7	11.3
	Missing	0	0.0
Uses olive oil at home?	No	2	3.2
	Yes	60	96.8
	Missing	0	0.0
Takes two yogurts and some cheese daily?	No	40	64.5
	Yes	22	35.5
	Missing	0	0.0
Takes sweets and candy several times every day?	No	32	51.6
	Yes	30	48.4
	Missing	0	0.0

**Table 5:** MOBAK scores expressed as mean ± standard deviation. Significance level *p* < 0.05.

**Score**	**Score T0** **(Mean ± SD)**	**Score T1** **(Mean ± SD)**	**Z**	***p***
*Score 1*	2.67 ± 1.78	4.28 ± 1.82	−7.57	0.000
*Score 2*	4.69 ± 1.96	5.83 ± 1.70	−5.81	0.000
*Score 3*	7.35 ± 3.09	10.11 ± 2.94	−7.69	0.000

T0 = first evaluation; T1 = second evaluation after 9 months.

## Data Availability

The datasets presented in this article are not readily available because the data are part of an ongoing study.
